# Highly Pathogenic Avian Influenza A(H5N8) Virus from Waterfowl, South Korea, 2014

**DOI:** 10.3201/eid2009.140390

**Published:** 2014-09

**Authors:** Keun Bon Ku, Eun Hye Park, Jung Yum, Ji An Kim, Seung Kyoo Oh, Sang Heui Seo

**Affiliations:** Chungnam National University College of Veterinary Medicine, Daejeon, South Korea

**Keywords:** avian influenza virus, H5N8, epidemiology, viruses, influenza, respiratory infections, genetic characterization, novel viruses, South Korea, waterfowl

**To the Editor:** To date, 18 hemagglutinin (HA) subtypes and 11 neuraminidase (NA) subtypes have been identified in influenza A viruses (*1*–*4*). Influenza A viruses containing HA subtypes 1–16 circulate in aquatic birds (*1*,*2*), whereas those harboring HA subtypes 17 and 18 are found in bats (*3*,*4*).

On January 18, 2014, the government of South Korea reported an outbreak of highly pathogenic avian influenza A(H5N8) virus in breeding ducks in the southern part of Jeollabuk-Do Province (*5*). More than 12 million poultry have since been culled, but the spread of the virus continues in duck and chicken farms. We report the genetic characterization of this virus.

On February 15, 2014, a total of 200 fecal samples were collected from waterfowl in the Pungse River in Chungnam Province, which is geographically close to Jeollabuk-Do Province. All samples were inoculated into hens’ eggs, and influenza A viruses were confirmed by PCR by using influenza A–specific nucleoprotein (NP) primers. We obtained 1 isolate, A/waterfowl/Korea/S005/2014 (H5N8), and sequenced the full regions of all 8 genes as described (*6*). These sequences were deposited into GenBank under accession nos. KJ511809–KJ511816. 

We conducted a BLAST search (http://blast.ncbi.nlm.nih.gov/Blast.cgi, http://platform.gisaid.org/epi3/frontend#4ead5c) to identify the closest gene sequences to those of A/waterfowl/Korea/S005/2014 (H5N8) (Table). Sequences for polymerase basic (PB) 2 (99% homology), HA (97% homology), and NP (99% homology) genes were closely related to those of A/wild duck/Shandong/628/2011 (H5N1). Sequences for PB1 (99% homology), polymerase acidic subunit (PA) (98% homology), matrix (M) (99% homology), and nonstructural (NS) (99% homology) genes were closely related to those of A/duck/Jiangsu/1-15/2011 (H4N2). Sequences for the NA (98% homology) gene were closely related to that of A/duck/Jiangsu/k1203/2010 (H5N8). Phylogenic analysis showed that all 8 genes of A/waterfowl/Korea/S005/2014 (H5N8) belonged to the Eurasian lineage, and that the HA gene clustered with clade 2.3.4 (Technical Appendix Figure 1).

**Table:** Nucleotide homology of genes of influenza virus strain A/waterfowl/Korea/S005/2014 (H5N8) to the closest related influenza virus strains*

Gene	Closest related virus strain	Nucleotide identity, %
PB2	A/wild duck/Shandong/628/2011 (H5N1)	99
PB1	A/duck/Jiangsu/1-15/2011 (H4N2)	99
PA	A/duck/Jiangsu/1-15/2011 (H4N2)	98
HA	A/wild duck/Shandong/628/2011 (H5N1)	97
NP	A/wild duck/Shandong/1/2011 (H5N1)	99
NA	A/duck/Jiangsu/k1203/2010 (H5N8)	98
M	A/duck/Jiangsu/1-15/2011 (H4N2)	99
NS	A/duck/Jiangsu/1-15/2011 (H4N2)	99

*PB, polymerase basic subunit; PA, polymerase acidic subunit; HA, hemagglutinin; NP, nucleoprotein; NA, neuraminidase; M, matrix; NS, nonstructural.

We further analyzed the amino acid sequences of the virus isolate (online Technical Appendix Table 1). Positions 138 and 160 of the HA protein (H3 numbering) contained an alanine (A) residue, which was previously found to be related to enhanced binding to the human influenza receptor (*7*). The connecting peptide of HA contained an insertion of 4 basic amino acids (arginine-arginine-arginine-lysine), which is the same as in the HA of A/duck/Korea/Buan2/2014 (H5N8), an isolate from a duck farm in South Korea (GenBank accession no. KJ413839.1–KJ413846.1). Aspartic acid was found in M1 at position 30 and alanine at position 215; this combination has been connected with increased virulence in mice (*8*). The NS1 sequence contained serine at position 42, which is related to the enhanced pathogenicity in mice, but a truncation of the amino acids at positions 218–230 that has been linked with reduced pathogenicity in mice (*9*) was not identified. Asparagine was identified at position 31 of M2, which is the same in M2 of A/duck/Korea/Buan2/2014 (H5N8) and confers resistance to amantadine and rimantadine (*10*).

Because all 8 genes of A/waterfowl/Korea/S005/2014 (H5N8) are closely related to those of the A/duck/Korea/Buan2/2014 (H5N8) isolate that was obtained from a duck farm, it is likely that A/waterfowl/Korea/S005/2014 (H5N8) originated from infected waterfowl that had visited poultry on an infected farm (Technical Appendix Figure 1). Our laboratory has studied the feces of wild birds in Chungnam Province since 2009, surveying >20,000 fecal samples from wild birds in this area each year, but we had not previously isolated avian influenza A(H5N8) virus from any samples.

The genetic analysis of the A/waterfowl/Korea/S005/2014 (H5N8) isolate indicates that this novel strain may have been created by the reassortment of PB2, HA, and NP segments from H5N1-like avian influenza virus; PB1, PA, M, and NS segments from H4N2-like avian influenza virus; and NA segments from H5N8-like avian influenza virus (Technical Appendix Figure 2). Most genes of the virus we isolated are related to those of avian influenza viruses isolated in China, but the HA gene of A/waterfowl/Korea/S005/2014 (H5N8) showed only 97% homology to the closest HA gene in GenBank, which indicates that this gene may have been created in poultry in South Korea. To our knowledge, no outbreak of this virus in poultry farms in China has been reported, and we found no previous reports in the literature that migratory birds could carry the virus. Taken together, our data suggest that A/waterfowl/Korea/S005/2014 (H5N8) may have been reassorted in a duck farm in South Korea.

Technical Appendix Phylogenetic analysis, schematic diagram, and identification of amino acids of influenza virus strain A/waterfowl/Korea/S005/2014 (H5N8).
