# Supplementary material for: Highly Pathogenic Avian Influenza A(H5N8) Virus from Waterfowl, South Korea, 2014
Source: Emerg Infect Dis. 2014 Sep;20(9):1587–8. doi: 10.3201/eid2009.140390 (PMC4178401; doi:10.3201/eid2009.140390)
Supplement: Technical Appendix — Phylogenetic analysis, schematic diagram, and identification of amino acids of influenza virus strain A/waterfowl/Korea/S005/2014 (H5N8). [file 14-0390-Techapp-s1.pdf]

Article DOI: http://dx.doi.org/10.3201/eid2009.140390

Highly Pathogenic Avian Influenza A(H5N8) Virus from Waterfowl, South Korea, 2014

Technical Appendix

Technical Appendix Figure 1 (following pages). Phylogenetic analysis of PB2, PB1, PA, HA, NP, NA, M, and NS genes of influenza virus strain A/waterfowl/Korea/S005/2014 (H5N8) (indicated by triangles). The trees were constructed using the neighbor-joining method in MEGA5 (http://www.megasoftware.net) with 1,000 bootstrap replicates. Scale bars indicate nucleotide substitutions per site. The HA was rooted to A/Goose/Guangdong/1/1996. The clade of HA gene was determined by BLAST search (http://www.fludb.org/brc/h5n1Classifier.spg?method=ShowCleanInputPage&decorator=influenza). Underlines indicate recent H5N8 isolates. PB, polymerase basic subunit; PA, polymerase acidic subunit; HA, hemagglutinin; NP, nucleoprotein; NA, neuraminidase; M, matrix; NS, nonstructural.

PB2

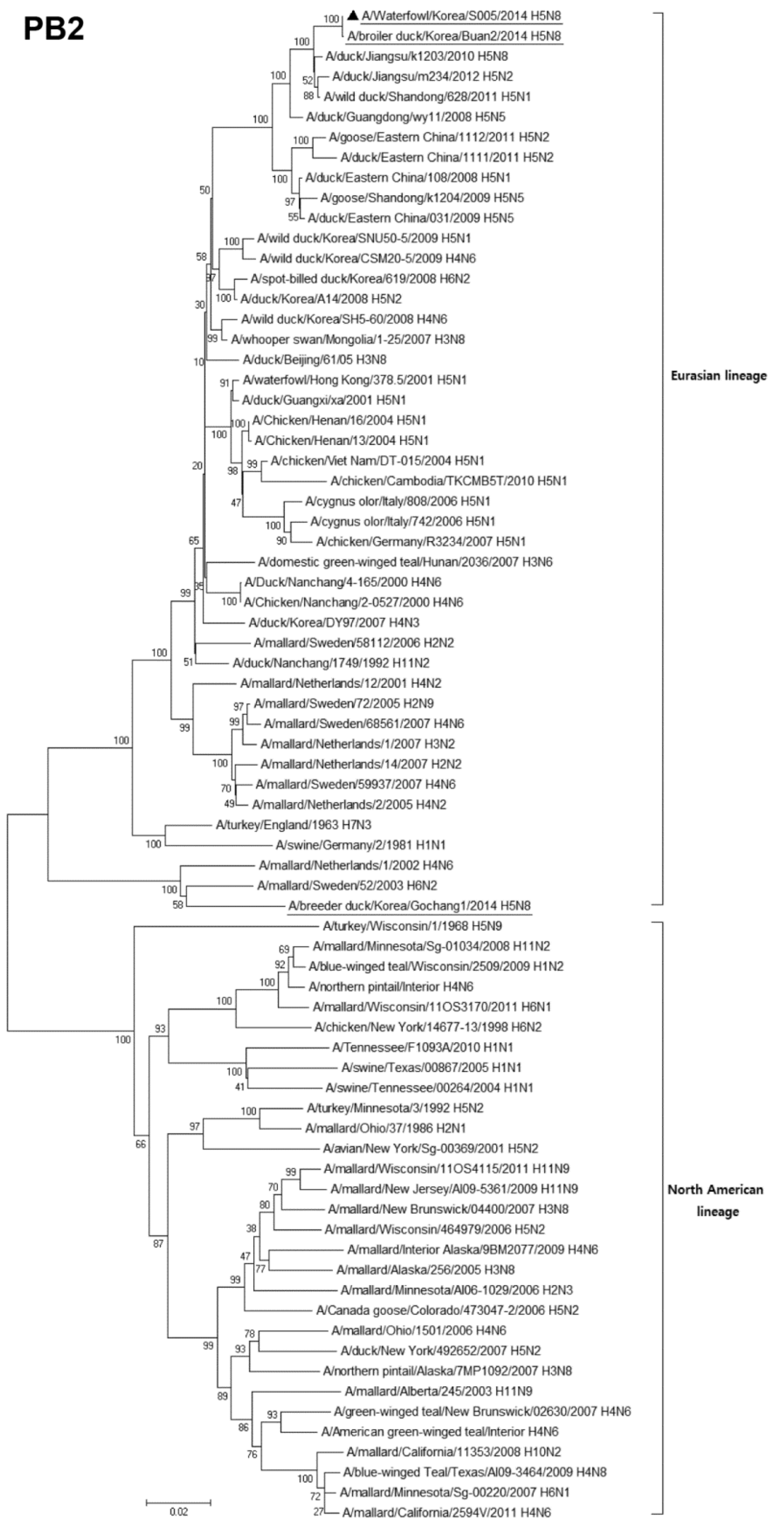
A/Waterfowl/Korea/S005/2014 H5N8
A/broiler duck/Korea/Buan2/2014 H5N8
A/duck/Jiangsu/k1203/2010 H5N8
A/duck/Jiangsu/m234/2012 H5N2
A/wild duck/Shandong/628/2011 H5N1
A/duck/Guangdong/wy11/2008 H5N5
A/goose/Eastern China/1112/2011 H5N2
A/duck/Eastern China/1111/2011 H5N2
A/duck/Eastern China/108/2008 H5N1
A/goose/Shandong/k1204/2009 H5N5
A/duck/Eastern China/031/2009 H5N5
A/wild duck/Korea/SNU50-5/2009 H5N1
A/wild duck/Korea/CSM20-5/2009 H4N6
A/spot-billed duck/Korea/619/2008 H6N2
A/duck/Korea/A14/2008 H5N2
A/wild duck/Korea/SH5-60/2008 H4N6
A/whooper swan/Mongolia/1-25/2007 H3N8
A/duck/Beijing/61/05 H3N8
A/waterfowl/Hong Kong/378.5/2001 H5N1
A/duck/Guangxi/xa/2001 H5N1
A/Chicken/Henan/16/2004 H5N1
A/Chicken/Henan/13/2004 H5N1
A/chicken/Viet Nam/DT-015/2004 H5N1
A/chicken/Cambodia/TKCMB5T/2010 H5N1
A/cygnus olor/Italy/808/2006 H5N1
A/cygnus olor/Italy/742/2006 H5N1
A/chicken/Germany/R3234/2007 H5N1
A/domestic green-winged teal/Hunan/2036/2007 H3N6
A/Duck/Nanchang/4-165/2000 H4N6
A/Chicken/Nanchang/2-0527/2000 H4N6
A/duck/Korea/DY97/2007 H4N3
A/mallard/Sweden/58112/2006 H2N2
A/duck/Nanchang/1749/1992 H11N2
A/mallard/Netherlands/12/2001 H4N2
A/mallard/Sweden/72/2005 H2N9
A/mallard/Sweden/68561/2007 H4N6
A/mallard/Netherlands/1/2007 H3N2
A/mallard/Netherlands/14/2007 H2N2
A/mallard/Sweden/59937/2007 H4N6
A/mallard/Netherlands/2/2005 H4N2
A/turkey/England/1963 H7N3
A/swine/Germany/2/1981 H1N1
A/mallard/Netherlands/1/2002 H4N6
A/mallard/Sweden/52/2003 H6N2
A/breeder duck/Korea/Gochang1/2014 H5N8
Eurasian lineage
A/turkey/Wisconsin/1/1968 H5N9
A/mallard/Minnesota/Sg-01034/2008 H11N2
A/blue-winged teal/Wisconsin/2509/2009 H1N2
A/northern pintail/Interior H4N6
A/mallard/Wisconsin/11OS3170/2011 H6N1
A/chicken/New York/14677-13/1998 H6N2
A/Tennessee/F1093A/2010 H1N1
A/swine/Texas/00867/2005 H1N1
A/swine/Tennessee/00264/2004 H1N1
A/turkey/Minnesota/3/1992 H5N2
A/mallard/Ohio/37/1986 H2N1
A/avian/New York/Sg-00369/2001 H5N2
A/mallard/Wisconsin/11OS4115/2011 H11N9
A/mallard/New Jersey/AI09-5361/2009 H11N9
A/mallard/New Brunswick/04400/2007 H3N8
A/mallard/Wisconsin/464979/2006 H5N2
A/mallard/Interior Alaska/9BM2077/2009 H4N6
A/mallard/Alaska/256/2005 H3N8
A/mallard/Minnesota/AI06-1029/2006 H2N3
A/Canada goose/Colorado/473047-2/2006 H5N2
A/mallard/Ohio/1501/2006 H4N6
A/duck/New York/492652/2007 H5N2
A/northern pintail/Alaska/7MP1092/2007 H3N8
A/mallard/Alberta/245/2003 H11N9
A/green-winged teal/New Brunswick/02630/2007 H4N6
A/American green-winged teal/Interior H4N6
A/mallard/California/11353/2008 H10N2
A/blue-winged Teal/Texas/AI09-3464/2009 H4N8
A/mallard/Minnesota/Sg-00220/2007 H6N1
A/mallard/California/2594V/2011 H4N6
North American lineage
0.02

PB1

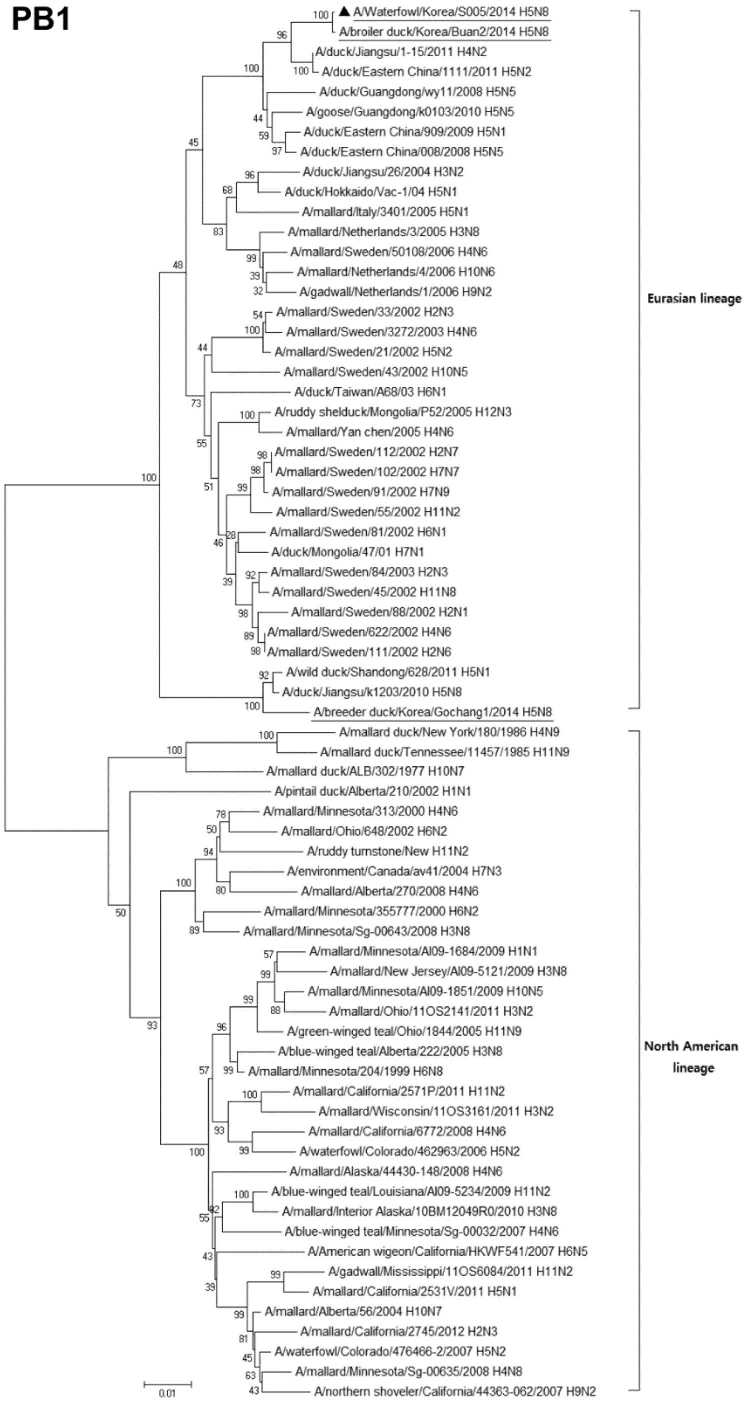
A/Waterfowl/Korea/S005/2014 H5N8
A/broiler duck/Korea/Buan2/2014 H5N8
A/duck/Jiangsu/1-15/2011 H4N2
A/duck/Eastern China/1111/2011 H5N2
A/duck/Guangdong/wy11/2008 H5N5
A/goose/Guangdong/k0103/2010 H5N5
A/duck/Eastern China/909/2009 H5N1
A/duck/Eastern China/008/2008 H5N5
A/duck/Jiangsu/26/2004 H3N2
A/duck/Hokkaido/Vac-1/04 H5N1
A/mallard/Italy/3401/2005 H5N1
A/mallard/Netherlands/3/2005 H3N8
A/mallard/Sweden/50108/2006 H4N6
A/mallard/Netherlands/4/2006 H10N6
A/gadwall/Netherlands/1/2006 H9N2
A/mallard/Sweden/33/2002 H2N3
A/mallard/Sweden/3272/2003 H4N6
A/mallard/Sweden/21/2002 H5N2
A/mallard/Sweden/43/2002 H10N5
A/duck/Taiwan/A68/03 H6N1
A/ruddy shelduck/Mongolia/P52/2005 H12N3
A/mallard/Yan chen/2005 H4N6
A/mallard/Sweden/112/2002 H2N7
A/mallard/Sweden/102/2002 H7N7
A/mallard/Sweden/91/2002 H7N9
A/mallard/Sweden/55/2002 H11N2
A/mallard/Sweden/81/2002 H6N1
A/duck/Mongolia/47/01 H7N1
A/mallard/Sweden/84/2003 H2N3
A/mallard/Sweden/45/2002 H11N8
A/mallard/Sweden/88/2002 H2N1
A/mallard/Sweden/622/2002 H4N6
A/mallard/Sweden/111/2002 H2N6
A/wild duck/Shandong/628/2011 H5N1
A/duck/Jiangsu/k1203/2010 H5N8
A/breeder duck/Korea/Gochang1/2014 H5N8
Eurasian lineage
A/mallard duck/New York/180/1986 H4N9
A/mallard duck/Tennessee/11457/1985 H11N9
A/mallard duck/ALB/302/1977 H10N7
A/pintail duck/Alberta/210/2002 H1N1
A/mallard/Minnesota/313/2000 H4N6
A/mallard/Ohio/648/2002 H6N2
A/ruddy turnstone/New H11N2
A/environment/Canada/av41/2004 H7N3
A/mallard/Alberta/270/2008 H4N6
A/mallard/Minnesota/355777/2000 H6N2
A/mallard/Minnesota/Sg-00643/2008 H3N8
A/mallard/Minnesota/AI09-1684/2009 H1N1
A/mallard/New Jersey/AI09-5121/2009 H3N8
A/mallard/Minnesota/AI09-1851/2009 H10N5
A/mallard/Ohio/11OS2141/2011 H3N2
A/green-winged teal/Ohio/1844/2005 H11N9
A/blue-winged teal/Alberta/222/2005 H3N8
A/mallard/Minnesota/204/1999 H6N8
A/mallard/California/2571P/2011 H11N2
A/mallard/Wisconsin/11OS3161/2011 H3N2
A/mallard/California/6772/2008 H4N6
A/waterfowl/Colorado/462963/2006 H5N2
A/mallard/Alaska/44430-148/2008 H4N6
A/blue-winged teal/Louisiana/AI09-5234/2009 H11N2
A/mallard/Interior Alaska/10BM12049R0/2010 H3N8
A/blue-winged teal/Minnesota/Sg-00032/2007 H4N6
A/American wigeon/California/HKWF541/2007 H6N5
A/gadwall/Mississippi/11OS6084/2011 H11N2
A/mallard/California/2531V/2011 H5N1
A/mallard/Alberta/56/2004 H10N7
A/mallard/California/2745/2012 H2N3
A/waterfowl/Colorado/476466-2/2007 H5N2
A/mallard/Minnesota/Sg-00635/2008 H4N8
A/northern shoveler/California/44363-062/2007 H9N2
North American lineage
0.01

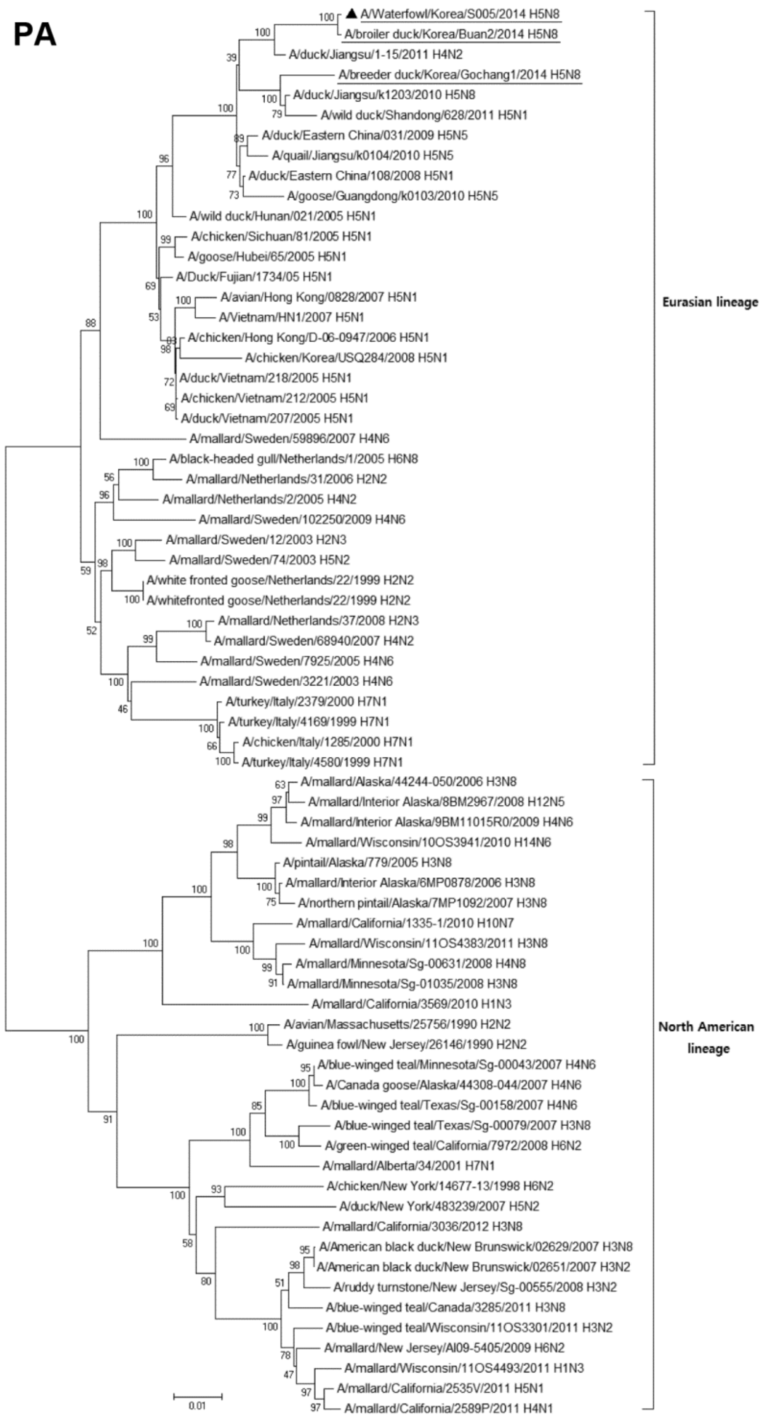
PA
A/Waterfowl/Korea/S005/2014 H5N8
A/broiler duck/Korea/Buan2/2014 H5N8
A/duck/Jiangsu/1-15/2011 H4N2
A/breeder duck/Korea/Gochang1/2014 H5N8
A/duck/Jiangsu/k1203/2010 H5N8
A/wild duck/Shandong/628/2011 H5N1
A/duck/Eastern China/031/2009 H5N5
A/quail/Jiangsu/k0104/2010 H5N5
A/duck/Eastern China/108/2008 H5N1
A/goose/Guangdong/k0103/2010 H5N5
A/wild duck/Hunan/021/2005 H5N1
A/chicken/Sichuan/81/2005 H5N1
A/goose/Hubei/65/2005 H5N1
A/Duck/Fujian/1734/05 H5N1
A/avian/Hong Kong/0828/2007 H5N1
A/Vietnam/HN1/2007 H5N1
A/chicken/Hong Kong/D-06-0947/2006 H5N1
A/chicken/Korea/USQ284/2008 H5N1
A/duck/Vietnam/218/2005 H5N1
A/chicken/Vietnam/212/2005 H5N1
A/duck/Vietnam/207/2005 H5N1
A/mallard/Sweden/59896/2007 H4N6
A/black-headed gull/Netherlands/1/2005 H6N8
A/mallard/Netherlands/31/2006 H2N2
A/mallard/Netherlands/2/2005 H4N2
A/mallard/Sweden/102250/2009 H4N6
A/mallard/Sweden/12/2003 H2N3
A/mallard/Sweden/74/2003 H5N2
A/white fronted goose/Netherlands/22/1999 H2N2
A/whitefronted goose/Netherlands/22/1999 H2N2
A/mallard/Netherlands/37/2008 H2N3
A/mallard/Sweden/68940/2007 H4N2
A/mallard/Sweden/7925/2005 H4N6
A/mallard/Sweden/3221/2003 H4N6
A/turkey/Italy/2379/2000 H7N1
A/turkey/Italy/4169/1999 H7N1
A/chicken/Italy/1285/2000 H7N1
A/turkey/Italy/4580/1999 H7N1
Eurasian lineage
A/mallard/Alaska/44244-050/2006 H3N8
A/mallard/Interior Alaska/8BM2967/2008 H12N5
A/mallard/Interior Alaska/9BM11015R0/2009 H4N6
A/mallard/Wisconsin/10OS3941/2010 H14N6
A/pintail/Alaska/779/2005 H3N8
A/mallard/Interior Alaska/6MP0878/2006 H3N8
A/northern pintail/Alaska/7MP1092/2007 H3N8
A/mallard/California/1335-1/2010 H10N7
A/mallard/Wisconsin/11OS4383/2011 H3N8
A/mallard/Minnesota/Sg-00631/2008 H4N8
A/mallard/Minnesota/Sg-01035/2008 H3N8
A/mallard/California/3569/2010 H1N3
A/avian/Massachusetts/25756/1990 H2N2
A/guinea fowl/New Jersey/26146/1990 H2N2
A/blue-winged teal/Minnesota/Sg-00043/2007 H4N6
A/Canada goose/Alaska/44308-044/2007 H4N6
A/blue-winged teal/Texas/Sg-00158/2007 H4N6
A/blue-winged teal/Texas/Sg-00079/2007 H3N8
A/green-winged teal/California/7972/2008 H6N2
A/mallard/Alberta/34/2001 H7N1
A/chicken/New York/14677-13/1998 H6N2
A/duck/New York/483239/2007 H5N2
A/mallard/California/3036/2012 H3N8
A/American black duck/New Brunswick/02629/2007 H3N8
A/American black duck/New Brunswick/02651/2007 H3N2
A/ruddy turnstone/New Jersey/Sg-00555/2008 H3N2
A/blue-winged teal/Canada/3285/2011 H3N8
A/blue-winged teal/Wisconsin/11OS3301/2011 H3N2
A/mallard/New Jersey/AI09-5405/2009 H6N2
A/mallard/Wisconsin/11OS4493/2011 H1N3
A/mallard/California/2535V/2011 H5N1
A/mallard/California/2589P/2011 H4N1
North American lineage
0.01

HA

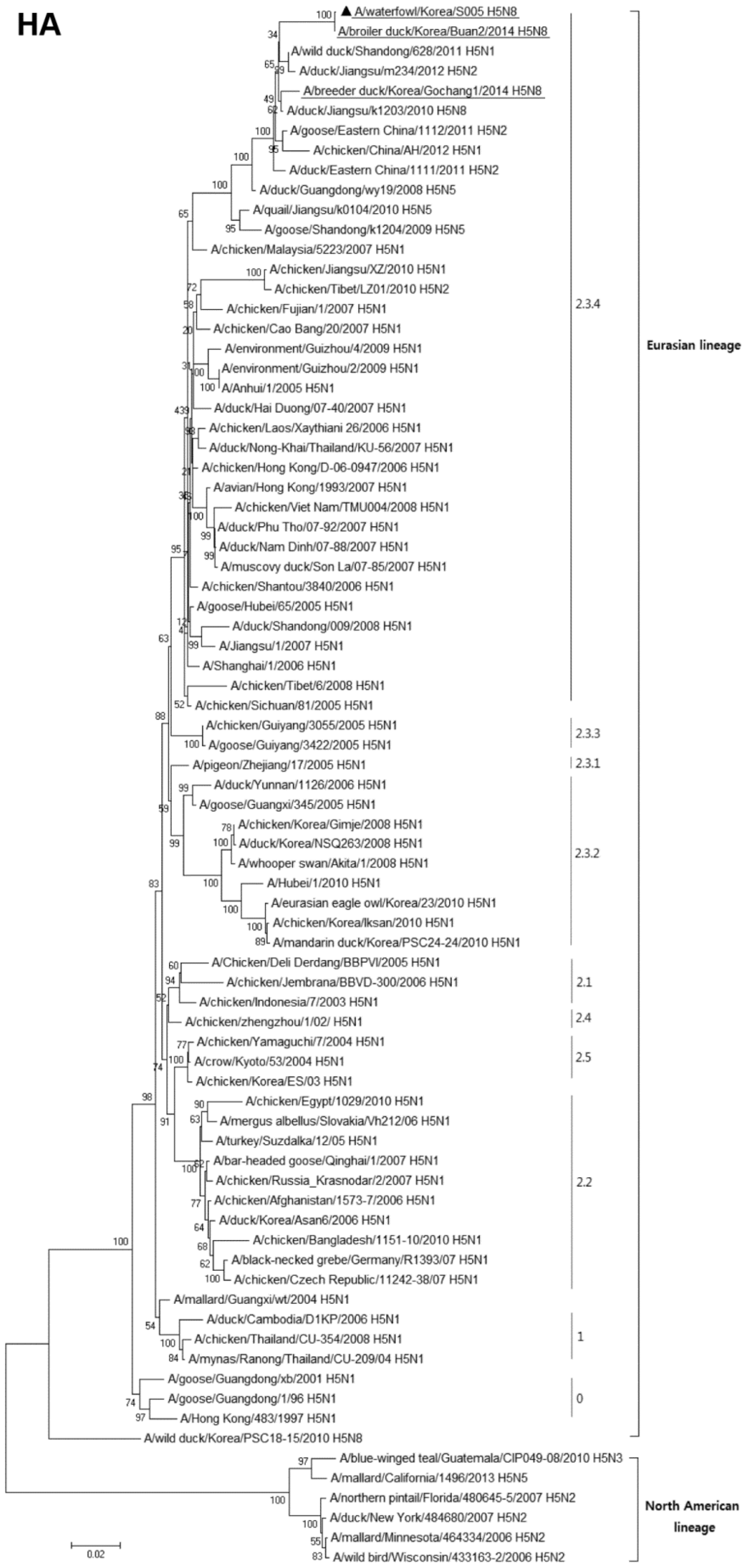

A/waterfowl/Korea/S005 H5N8
A/broiler duck/Korea/Buan2/2014 H5N8
A/wild duck/Shandong/628/2011 H5N1
A/duck/Jiangsu/m234/2012 H5N2
A/breeder duck/Korea/Gochang1/2014 H5N8
A/duck/Jiangsu/k1203/2010 H5N8
A/goose/Eastern China/1112/2011 H5N2
A/chicken/China/AH/2012 H5N1
A/duck/Eastern China/1111/2011 H5N2
A/duck/Guangdong/wy19/2008 H5N5
A/quail/Jiangsu/k0104/2010 H5N5
A/goose/Shandong/k1204/2009 H5N5
A/chicken/Malaysia/5223/2007 H5N1
A/chicken/Jiangsu/XZ/2010 H5N1
A/chicken/Tibet/LZ01/2010 H5N2
A/chicken/Fujian/1/2007 H5N1
A/chicken/Cao Bang/20/2007 H5N1
A/environment/Guizhou/4/2009 H5N1
A/environment/Guizhou/2/2009 H5N1
A/Anhui/1/2005 H5N1
A/duck/Hai Duong/07-40/2007 H5N1
A/chicken/Laos/Xaythiani 26/2006 H5N1
A/duck/Nong-Khai/Thailand/KU-56/2007 H5N1
A/chicken/Hong Kong/D-06-0947/2006 H5N1
A/avian/Hong Kong/1993/2007 H5N1
A/chicken/Viet Nam/TMU004/2008 H5N1
A/duck/Phu Tho/07-92/2007 H5N1
A/duck/Nam Dinh/07-88/2007 H5N1
A/muscovy duck/Son La/07-85/2007 H5N1
A/chicken/Shantou/3840/2006 H5N1
A/goose/Hubei/65/2005 H5N1
A/duck/Shandong/009/2008 H5N1
A/Jiangsu/1/2007 H5N1
A/Shanghai/1/2006 H5N1
A/chicken/Tibet/6/2008 H5N1
A/chicken/Sichuan/81/2005 H5N1
2.3.4
A/chicken/Guiyang/3055/2005 H5N1
A/goose/Guiyang/3422/2005 H5N1
2.3.3
A/pigeon/Zhejiang/17/2005 H5N1
2.3.1
A/duck/Yunnan/1126/2006 H5N1
A/goose/Guangxi/345/2005 H5N1
A/chicken/Korea/Gimje/2008 H5N1
A/duck/Korea/NSQ263/2008 H5N1
A/whooper swan/Akita/1/2008 H5N1
A/Hubei/1/2010 H5N1
A/eurasian eagle owl/Korea/23/2010 H5N1
A/chicken/Korea/Iksan/2010 H5N1
A/mandarin duck/Korea/PSC24-24/2010 H5N1
2.3.2
A/Chicken/Deli Derdang/BBPVI/2005 H5N1
A/chicken/Jembrana/BBVD-300/2006 H5N1
A/chicken/Indonesia/7/2003 H5N1
2.1
A/chicken/zhengzhou/1/02/ H5N1
2.4
A/chicken/Yamaguchi/7/2004 H5N1
A/crow/Kyoto/53/2004 H5N1
A/chicken/Korea/ES/03 H5N1
2.5
A/chicken/Egypt/1029/2010 H5N1
A/mergus albellus/Slovakia/Vh212/06 H5N1
A/turkey/Suzdalka/12/05 H5N1
A/bar-headed goose/Qinghai/1/2007 H5N1
A/chicken/Russia_Krasnodar/2/2007 H5N1
A/chicken/Afghanistan/1573-7/2006 H5N1
A/duck/Korea/Asan6/2006 H5N1
A/chicken/Bangladesh/1151-10/2010 H5N1
A/black-necked grebe/Germany/R1393/07 H5N1
A/chicken/Czech Republic/11242-38/07 H5N1
2.2
A/mallard/Guangxi/wt/2004 H5N1
A/duck/Cambodia/D1KP/2006 H5N1
A/chicken/Thailand/CU-354/2008 H5N1
A/mynas/Ranong/Thailand/CU-209/04 H5N1
1
A/goose/Guangdong/xb/2001 H5N1
A/goose/Guangdong/1/96 H5N1
A/Hong Kong/483/1997 H5N1
0
A/wild duck/Korea/PSC18-15/2010 H5N8
Eurasian lineage
A/blue-winged teal/Guatemala/CIP049-08/2010 H5N3
A/mallard/California/1496/2013 H5N5
A/northern pintail/Florida/480645-5/2007 H5N2
A/duck/New York/484680/2007 H5N2
A/mallard/Minnesota/464334/2006 H5N2
A/wild bird/Wisconsin/433163-2/2006 H5N2
North American lineage
0.02

NP

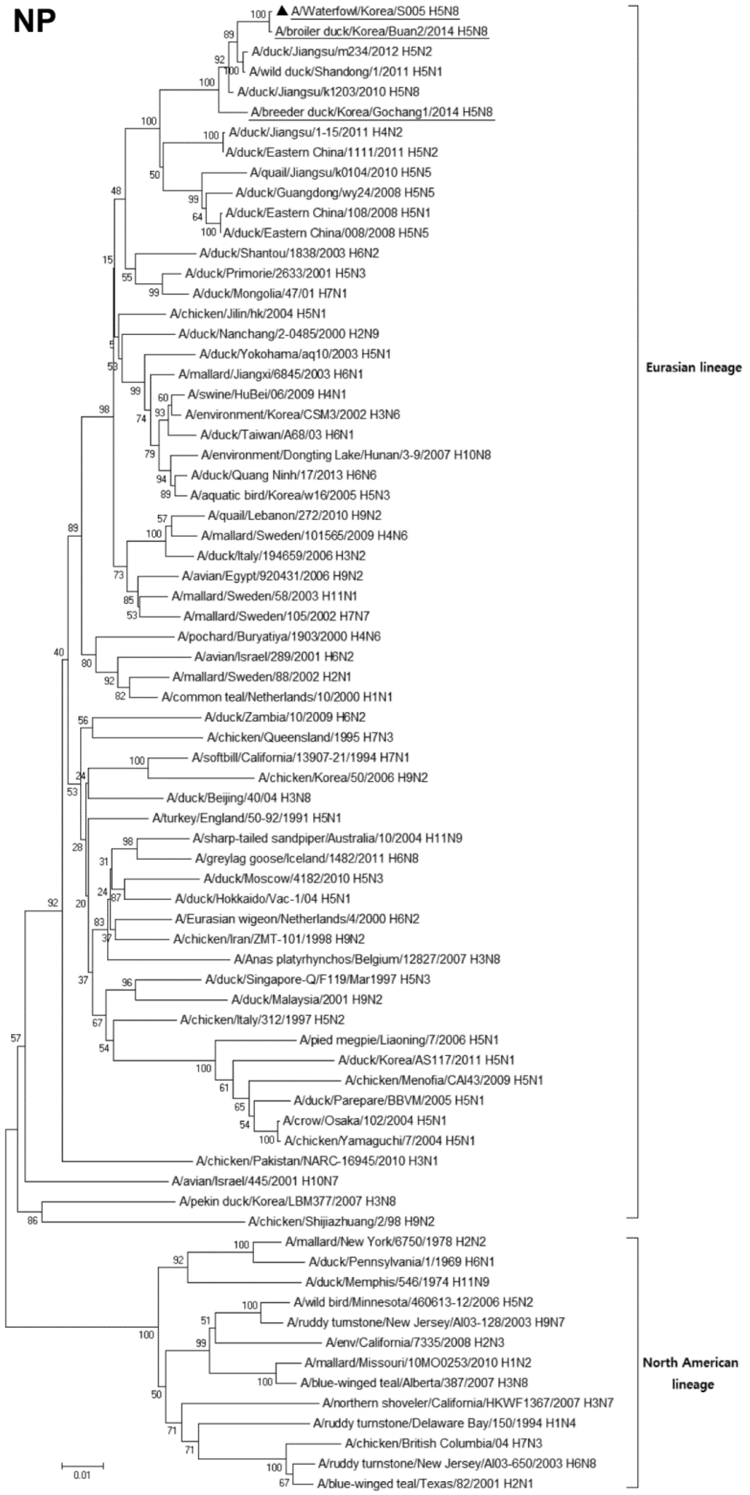

A/Waterfowl/Korea/S005 H5N8
A/broiler duck/Korea/Buan2/2014 H5N8
A/duck/Jiangsu/m234/2012 H5N2
A/wild duck/Shandong/1/2011 H5N1
A/duck/Jiangsu/k1203/2010 H5N8
A/breeder duck/Korea/Gochang1/2014 H5N8
A/duck/Jiangsu/1-15/2011 H4N2
A/duck/Eastern China/1111/2011 H5N2
A/quail/Jiangsu/k0104/2010 H5N5
A/duck/Guangdong/wy24/2008 H5N5
A/duck/Eastern China/108/2008 H5N1
A/duck/Eastern China/008/2008 H5N5
A/duck/Shantou/1838/2003 H6N2
A/duck/Primorie/2633/2001 H5N3
A/duck/Mongolia/47/01 H7N1
A/chicken/Jilin/hk/2004 H5N1
A/duck/Nanchang/2-0485/2000 H2N9
A/duck/Yokohama/aq10/2003 H5N1
A/mallard/Jiangxi/6845/2003 H6N1
A/swine/HuBei/06/2009 H4N1
A/environment/Korea/CSM3/2002 H3N6
A/duck/Taiwan/A68/03 H6N1
A/environment/Dongting Lake/Hunan/3-9/2007 H10N8
A/duck/Quang Ninh/17/2013 H6N6
A/aquatic bird/Korea/w16/2005 H5N3
A/quail/Lebanon/272/2010 H9N2
A/mallard/Sweden/101565/2009 H4N6
A/duck/Italy/194659/2006 H3N2
A/avian/Egypt/920431/2006 H9N2
A/mallard/Sweden/58/2003 H11N1
A/mallard/Sweden/105/2002 H7N7
A/pochard/Buryatiya/1903/2000 H4N6
A/avian/Israel/289/2001 H6N2
A/mallard/Sweden/88/2002 H2N1
A/common teal/Netherlands/10/2000 H1N1
A/duck/Zambia/10/2009 H6N2
A/chicken/Queensland/1995 H7N3
A/softbill/California/13907-21/1994 H7N1
A/chicken/Korea/50/2006 H9N2
A/duck/Beijing/40/04 H3N8
A/turkey/England/50-92/1991 H5N1
A/sharp-tailed sandpiper/Australia/10/2004 H11N9
A/greylag goose/Iceland/1482/2011 H6N8
A/duck/Moscow/4182/2010 H5N3
A/duck/Hokkaido/Vac-1/04 H5N1
A/Eurasian wigeon/Netherlands/4/2000 H6N2
A/chicken/Iran/ZMT-101/1998 H9N2
A/Anas platyrhynchos/Belgium/12827/2007 H3N8
A/duck/Singapore-Q/F119/Mar1997 H5N3
A/duck/Malaysia/2001 H9N2
A/chicken/Italy/312/1997 H5N2
A/pied megpie/Liaoning/7/2006 H5N1
A/duck/Korea/AS117/2011 H5N1
A/chicken/Menofia/CAI43/2009 H5N1
A/duck/Parepare/BBVM/2005 H5N1
A/crow/Osaka/102/2004 H5N1
A/chicken/Yamaguchi/7/2004 H5N1
A/chicken/Pakistan/NARC-16945/2010 H3N1
A/avian/Israel/445/2001 H10N7
A/pekin duck/Korea/LBM377/2007 H3N8
A/chicken/Shijiazhuang/2/98 H9N2
Eurasian lineage
A/mallard/New York/6750/1978 H2N2
A/duck/Pennsylvania/1/1969 H6N1
A/duck/Memphis/546/1974 H11N9
A/wild bird/Minnesota/460613-12/2006 H5N2
A/ruddy turnstone/New Jersey/AI03-128/2003 H9N7
A/env/California/7335/2008 H2N3
A/mallard/Missouri/10MO0253/2010 H1N2
A/blue-winged teal/Alberta/387/2007 H3N8
A/northern shoveler/California/HKWF1367/2007 H3N7
A/ruddy turnstone/Delaware Bay/150/1994 H1N4
A/chicken/British Columbia/04 H7N3
A/ruddy turnstone/New Jersey/AI03-650/2003 H6N8
A/blue-winged teal/Texas/82/2001 H2N1
North American lineage
0.01

NA

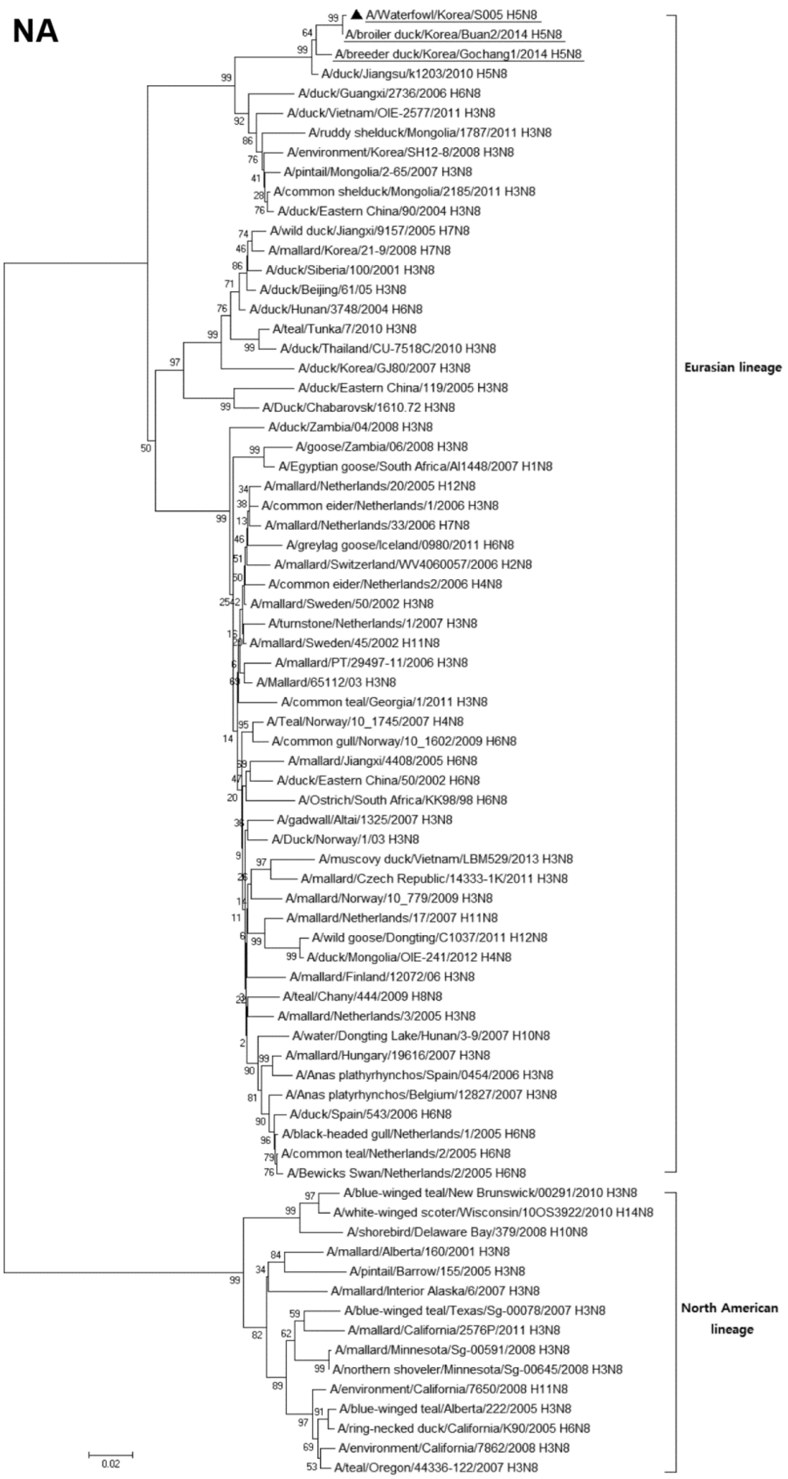
A/Waterfowl/Korea/S005 H5N8
A/broiler duck/Korea/Buan2/2014 H5N8
A/breeder duck/Korea/Gochang1/2014 H5N8
A/duck/Jiangsu/k1203/2010 H5N8
A/duck/Guangxi/2736/2006 H6N8
A/duck/Vietnam/OIE-2577/2011 H3N8
A/ruddy shelduck/Mongolia/1787/2011 H3N8
A/environment/Korea/SH12-8/2008 H3N8
A/pintail/Mongolia/2-65/2007 H3N8
A/common shelduck/Mongolia/2185/2011 H3N8
A/duck/Eastern China/90/2004 H3N8
A/wild duck/Jiangxi/9157/2005 H7N8
A/mallard/Korea/21-9/2008 H7N8
A/duck/Siberia/100/2001 H3N8
A/duck/Beijing/61/05 H3N8
A/duck/Hunan/3748/2004 H6N8
A/teal/Tunka/7/2010 H3N8
A/duck/Thailand/CU-7518C/2010 H3N8
A/duck/Korea/GJ80/2007 H3N8
A/duck/Eastern China/119/2005 H3N8
A/Duck/Chabarovsk/1610.72 H3N8
A/duck/Zambia/04/2008 H3N8
A/goose/Zambia/06/2008 H3N8
A/Egyptian goose/South Africa/Al1448/2007 H1N8
A/mallard/Netherlands/20/2005 H12N8
A/common eider/Netherlands/1/2006 H3N8
A/mallard/Netherlands/33/2006 H7N8
A/greylag goose/Iceland/0980/2011 H6N8
A/mallard/Switzerland/WV4060057/2006 H2N8
A/common eider/Netherlands2/2006 H4N8
A/mallard/Sweden/50/2002 H3N8
A/turnstone/Netherlands/1/2007 H3N8
A/mallard/Sweden/45/2002 H11N8
A/mallard/PT/29497-11/2006 H3N8
A/Mallard/65112/03 H3N8
A/common teal/Georgia/1/2011 H3N8
A/Teal/Norway/10_1745/2007 H4N8
A/common gull/Norway/10_1602/2009 H6N8
A/mallard/Jiangxi/4408/2005 H6N8
A/duck/Eastern China/50/2002 H6N8
A/Ostrich/South Africa/KK98/98 H6N8
A/gadwall/Altai/1325/2007 H3N8
A/Duck/Norway/1/03 H3N8
A/muscovy duck/Vietnam/LBM529/2013 H3N8
A/mallard/Czech Republic/14333-1K/2011 H3N8
A/mallard/Norway/10_779/2009 H3N8
A/mallard/Netherlands/17/2007 H11N8
A/wild goose/Dongting/C1037/2011 H12N8
A/duck/Mongolia/OIE-241/2012 H4N8
A/mallard/Finland/12072/06 H3N8
A/teal/Chany/444/2009 H8N8
A/mallard/Netherlands/3/2005 H3N8
A/water/Dongting Lake/Hunan/3-9/2007 H10N8
A/mallard/Hungary/19616/2007 H3N8
A/Anas plathyrhynchos/Spain/0454/2006 H3N8
A/Anas platyrhynchos/Belgium/12827/2007 H3N8
A/duck/Spain/543/2006 H6N8
A/black-headed gull/Netherlands/1/2005 H6N8
A/common teal/Netherlands/2/2005 H6N8
A/Bewicks Swan/Netherlands/2/2005 H6N8
Eurasian lineage
A/blue-winged teal/New Brunswick/00291/2010 H3N8
A/white-winged scoter/Wisconsin/10OS3922/2010 H14N8
A/shorebird/Delaware Bay/379/2008 H10N8
A/mallard/Alberta/160/2001 H3N8
A/pintail/Barrow/155/2005 H3N8
A/mallard/Interior Alaska/6/2007 H3N8
A/blue-winged teal/Texas/Sg-00078/2007 H3N8
A/mallard/California/2576P/2011 H3N8
A/mallard/Minnesota/Sg-00591/2008 H3N8
A/northern shoveler/Minnesota/Sg-00645/2008 H3N8
A/environment/California/7650/2008 H11N8
A/blue-winged teal/Alberta/222/2005 H3N8
A/ring-necked duck/California/K90/2005 H6N8
A/environment/California/7862/2008 H3N8
A/teal/Oregon/44336-122/2007 H3N8
North American lineage
0.02

M

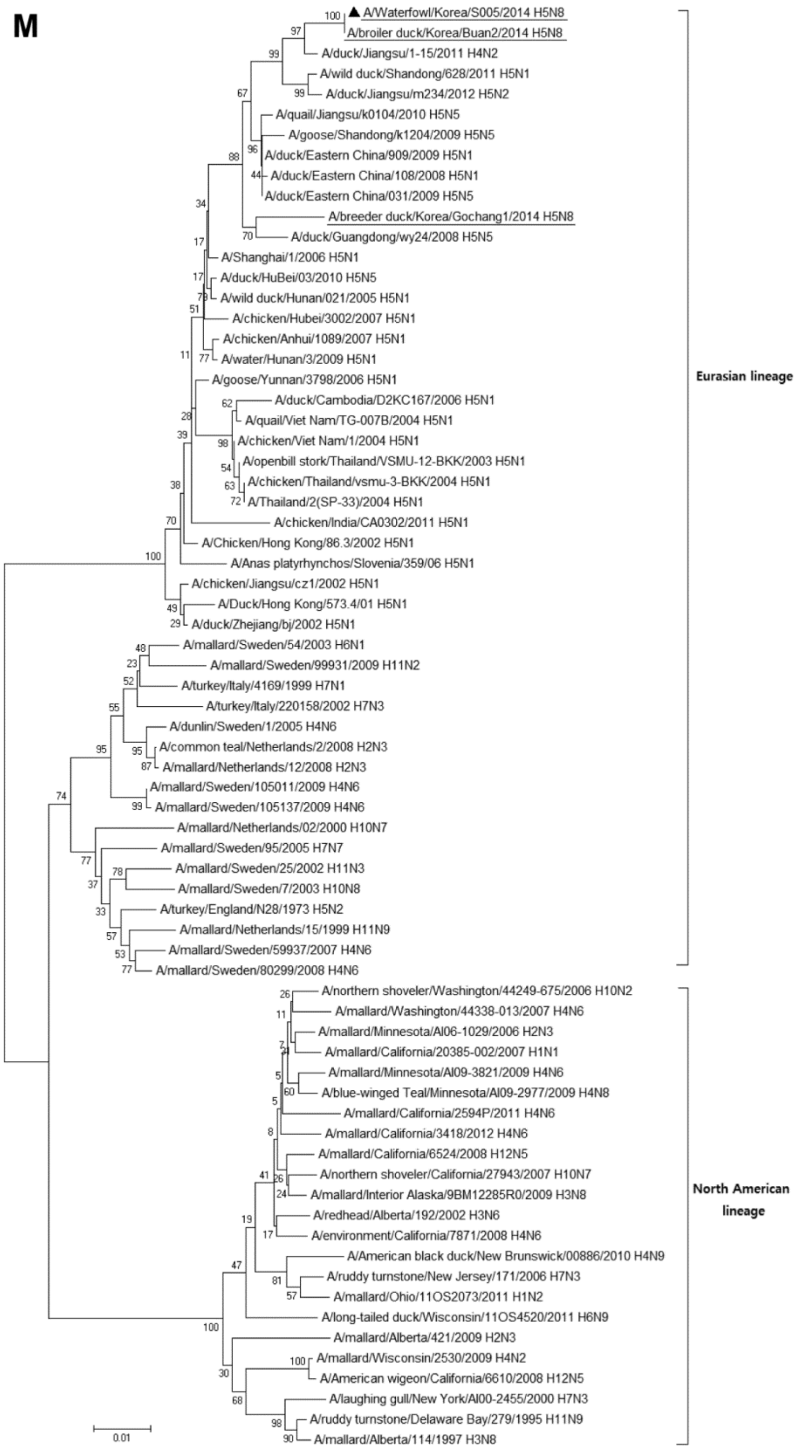

A/Waterfowl/Korea/S005/2014 H5N8
A/broiler duck/Korea/Buan2/2014 H5N8
A/duck/Jiangsu/1-15/2011 H4N2
A/wild duck/Shandong/628/2011 H5N1
A/duck/Jiangsu/m234/2012 H5N2
A/quail/Jiangsu/k0104/2010 H5N5
A/goose/Shandong/k1204/2009 H5N5
A/duck/Eastern China/909/2009 H5N1
A/duck/Eastern China/108/2008 H5N1
A/duck/Eastern China/031/2009 H5N5
A/breeder duck/Korea/Gochang1/2014 H5N8
A/duck/Guangdong/wy24/2008 H5N5
A/Shanghai/1/2006 H5N1
A/duck/HuBei/03/2010 H5N5
A/wild duck/Hunan/021/2005 H5N1
A/chicken/Hubei/3002/2007 H5N1
A/chicken/Anhui/1089/2007 H5N1
A/water/Hunan/3/2009 H5N1
A/goose/Yunnan/3798/2006 H5N1
A/duck/Cambodia/D2KC167/2006 H5N1
A/quail/Viet Nam/TG-007B/2004 H5N1
A/chicken/Viet Nam/1/2004 H5N1
A/openbill stork/Thailand/VSMU-12-BKK/2003 H5N1
A/chicken/Thailand/vsmu-3-BKK/2004 H5N1
A/Thailand/2(SP-33)/2004 H5N1
A/chicken/India/CA0302/2011 H5N1
A/Chicken/Hong Kong/86.3/2002 H5N1
A/Anas platyrhynchos/Slovenia/359/06 H5N1
A/chicken/Jiangsu/cz1/2002 H5N1
A/Duck/Hong Kong/573.4/01 H5N1
A/duck/Zhejiang/bj/2002 H5N1
A/mallard/Sweden/54/2003 H6N1
A/mallard/Sweden/99931/2009 H11N2
A/turkey/Italy/4169/1999 H7N1
A/turkey/Italy/220158/2002 H7N3
A/dunlin/Sweden/1/2005 H4N6
A/common teal/Netherlands/2/2008 H2N3
A/mallard/Netherlands/12/2008 H2N3
A/mallard/Sweden/105011/2009 H4N6
A/mallard/Sweden/105137/2009 H4N6
A/mallard/Netherlands/02/2000 H10N7
A/mallard/Sweden/95/2005 H7N7
A/mallard/Sweden/25/2002 H11N3
A/mallard/Sweden/7/2003 H10N8
A/turkey/England/N28/1973 H5N2
A/mallard/Netherlands/15/1999 H11N9
A/mallard/Sweden/59937/2007 H4N6
A/mallard/Sweden/80299/2008 H4N6
Eurasian lineage
A/northern shoveler/Washington/44249-675/2006 H10N2
A/mallard/Washington/44338-013/2007 H4N6
A/mallard/Minnesota/AI06-1029/2006 H2N3
A/mallard/California/20385-002/2007 H1N1
A/mallard/Minnesota/AI09-3821/2009 H4N6
A/blue-winged Teal/Minnesota/AI09-2977/2009 H4N8
A/mallard/California/2594P/2011 H4N6
A/mallard/California/3418/2012 H4N6
A/mallard/California/6524/2008 H12N5
A/northern shoveler/California/27943/2007 H10N7
A/mallard/Interior Alaska/9BM12285R0/2009 H3N8
A/redhead/Alberta/192/2002 H3N6
A/environment/California/7871/2008 H4N6
A/American black duck/New Brunswick/00886/2010 H4N9
A/ruddy turnstone/New Jersey/171/2006 H7N3
A/mallard/Ohio/11OS2073/2011 H1N2
A/long-tailed duck/Wisconsin/11OS4520/2011 H6N9
A/mallard/Alberta/421/2009 H2N3
A/mallard/Wisconsin/2530/2009 H4N2
A/American wigeon/California/6610/2008 H12N5
A/laughing gull/New York/AI00-2455/2000 H7N3
A/ruddy turnstone/Delaware Bay/279/1995 H11N9
A/mallard/Alberta/114/1997 H3N8
North American lineage
0.01

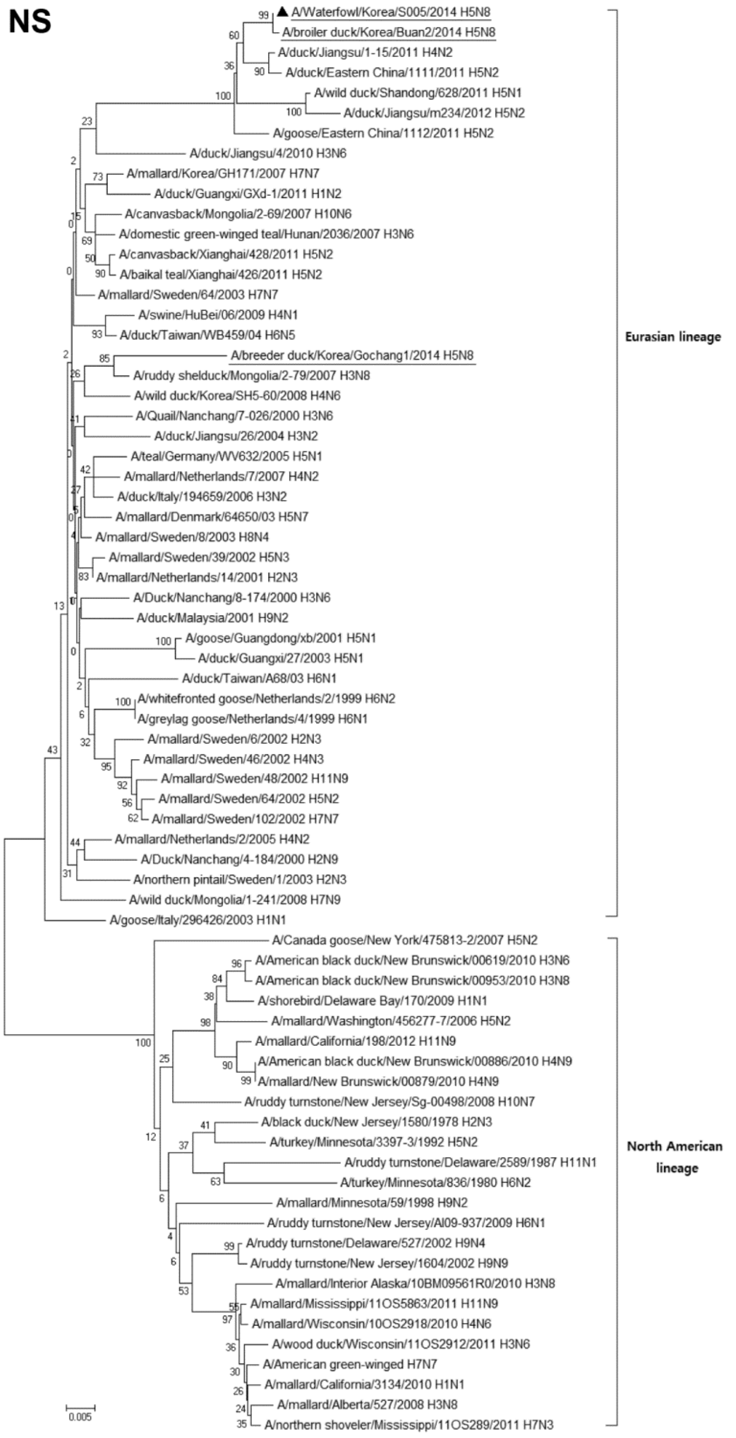
NS
A/Waterfowl/Korea/S005/2014 H5N8
A/broiler duck/Korea/Buan2/2014 H5N8
A/duck/Jiangsu/1-15/2011 H4N2
A/duck/Eastern China/1111/2011 H5N2
A/wild duck/Shandong/628/2011 H5N1
A/duck/Jiangsu/m234/2012 H5N2
A/goose/Eastern China/1112/2011 H5N2
A/duck/Jiangsu/4/2010 H3N6
A/mallard/Korea/GH171/2007 H7N7
A/duck/Guangxi/GXd-1/2011 H1N2
A/canvasback/Mongolia/2-69/2007 H10N6
A/domestic green-winged teal/Hunan/2036/2007 H3N6
A/canvasback/Xianghai/428/2011 H5N2
A/baikal teal/Xianghai/426/2011 H5N2
A/mallard/Sweden/64/2003 H7N7
A/swine/HuBei/06/2009 H4N1
A/duck/Taiwan/WB459/04 H6N5
A/breeder duck/Korea/Gochang1/2014 H5N8
A/ruddy shelduck/Mongolia/2-79/2007 H3N8
A/wild duck/Korea/SH5-60/2008 H4N6
A/Quail/Nanchang/7-026/2000 H3N6
A/duck/Jiangsu/26/2004 H3N2
A/teal/Germany/WV632/2005 H5N1
A/mallard/Netherlands/7/2007 H4N2
A/duck/Italy/194659/2006 H3N2
A/mallard/Denmark/64650/03 H5N7
A/mallard/Sweden/8/2003 H8N4
A/mallard/Sweden/39/2002 H5N3
A/mallard/Netherlands/14/2001 H2N3
A/Duck/Nanchang/8-174/2000 H3N6
A/duck/Malaysia/2001 H9N2
A/goose/Guangdong/xb/2001 H5N1
A/duck/Guangxi/27/2003 H5N1
A/duck/Taiwan/A68/03 H6N1
A/whitefronted goose/Netherlands/2/1999 H6N2
A/greylag goose/Netherlands/4/1999 H6N1
A/mallard/Sweden/6/2002 H2N3
A/mallard/Sweden/46/2002 H4N3
A/mallard/Sweden/48/2002 H11N9
A/mallard/Sweden/64/2002 H5N2
A/mallard/Sweden/102/2002 H7N7
A/mallard/Netherlands/2/2005 H4N2
A/Duck/Nanchang/4-184/2000 H2N9
A/northern pintail/Sweden/1/2003 H2N3
A/wild duck/Mongolia/1-241/2008 H7N9
A/goose/Italy/296426/2003 H1N1
Eurasian lineage
A/Canada goose/New York/475813-2/2007 H5N2
A/American black duck/New Brunswick/00619/2010 H3N6
A/American black duck/New Brunswick/00953/2010 H3N8
A/shorebird/Delaware Bay/170/2009 H1N1
A/mallard/Washington/456277-7/2006 H5N2
A/mallard/California/198/2012 H11N9
A/American black duck/New Brunswick/00886/2010 H4N9
A/mallard/New Brunswick/00879/2010 H4N9
A/ruddy turnstone/New Jersey/Sg-00498/2008 H10N7
A/black duck/New Jersey/1580/1978 H2N3
A/turkey/Minnesota/3397-3/1992 H5N2
A/ruddy turnstone/Delaware/2589/1987 H11N1
A/turkey/Minnesota/836/1980 H6N2
A/mallard/Minnesota/59/1998 H9N2
A/ruddy turnstone/New Jersey/AI09-937/2009 H6N1
A/ruddy turnstone/Delaware/527/2002 H9N4
A/ruddy turnstone/New Jersey/1604/2002 H9N9
A/mallard/Interior Alaska/10BM09561R0/2010 H3N8
A/mallard/Mississippi/11OS5863/2011 H11N9
A/mallard/Wisconsin/10OS2918/2010 H4N6
A/wood duck/Wisconsin/11OS2912/2011 H3N6
A/American green-winged H7N7
A/mallard/California/3134/2010 H1N1
A/mallard/Alberta/527/2008 H3N8
A/northern shoveler/Mississippi/11OS289/2011 H7N3
North American lineage
0.005

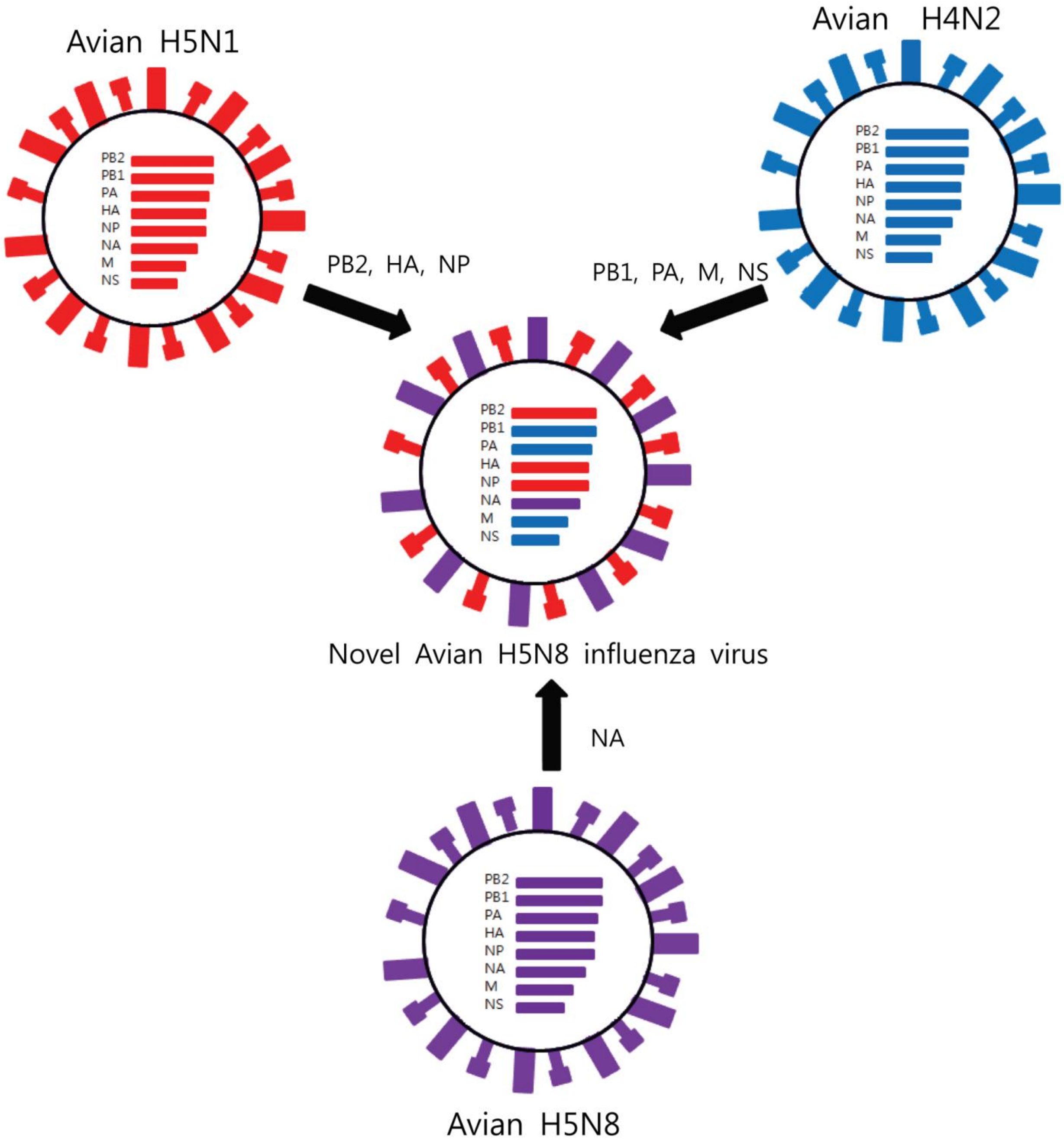

Technical Appendix Figure 2. Schematic diagram of influenza virus strain A/waterfowl/Korea/S005/2014 (H5N8). Novel highly pathogenic avian influenza virus is likely to be created by genes from 3 avian influenza viruses. PB, polymerase basic subunit; PA, polymerase acidic subunit; HA, hemagglutinin; NP, nucleoprotein; NA, neuraminidase; M, matrix; NS, nonstructural.

Technical Appendix Table. Identification of amino acids of influenza virus strain A/waterfowl/Korea/S005/2014 (H5N8) involved in binding to human-type influenza receptor, enhancing antiviral drugs, and causing pathogenesis in poultry and mammals

Viral protein*	Amino acid position	A/waterfowl/Korea/S005/2014 (H5N8)†	Comments
PB2	627	E	E627K: adaptation to mammalian host
HA	138 (H3 numbering)	A	S138A: Increased binding to human-type influenza receptor
	160 (H3 numbering)	A	T160A: N-glycosylation loss and increased binding to human-type influenza receptor
	226 (H3 numbering)	Q	Q226L: Increased binding to human-type influenza receptor
	228 (H3 numbering)	G	G228S: Increased binding to human-type influenza receptor
	339-348	RE RRRK R/GLF	Polybasic amino acid insertion: high pathogenesis in poultry and mammals
NA	69-72 (N9 numbering)	No deletion	Deletion of amino acids 69-73:Increased pathogenesis in mice
	292 (N2 numbering)	R	R292K: Resistance to oseltamivir and zanamivir
M1	30	D	N30D: Increased pathogenesis in mice
	215	A	T215A: Increased pathogenesis in mice
M2	31	N	S31N: Resistance to amantadine and rimantadine
NS1	42	S	P42S: Increased pathogenesis in mice
	218-230	No truncation	Lack of PDZ domain binding motif: reduced pathogenesis in mice

*PB, polymerase basic subunit; PA, polymerase acidic subunit; HA, hemagglutinin; NP, nucleoprotein; NA, neuraminidase; M, matrix; NS, nonstructural.

†A, alanine; D, aspartic acid; E, glutamic acid; F, phenylalanine; G, glycine; K, lysine; L, leucine; N, asparagine; P, proline; Q, glutamine; R, arginine; S, serine; T, threonine.
